# Experimental Evolution of *Trichoderma citrinoviride* for Faster Deconstruction of Cellulose

**DOI:** 10.1371/journal.pone.0147024

**Published:** 2016-01-28

**Authors:** Hui Lin, Michael Travisano, Romas J. Kazlauskas

**Affiliations:** 1 The Biotechnology Institute, University of Minnesota, Twin Cities, Minnesota, United States of America; 2 Department of Ecology Evolution and Behavior, University of Minnesota, Twin Cities, Minnesota, United States of America; 3 Department of Biochemistry, Molecular Biology and Biophysics, University of Minnesota, Twin Cities, Minnesota, United States of America; The University of Wisconsin - Madison, UNITED STATES

## Abstract

Engineering faster cellulose deconstruction is difficult because it is a complex, cooperative, multi-enzyme process. Here we use experimental evolution to select for populations of *Trichoderma citrinoviride* that deconstruct up to five-fold more cellulose. Ten replicate populations of *T*. *citrinoviride* were selected for growth on filter paper by serial culture. After 125 periods of growth and transfer to fresh media, the filter paper deconstruction increased an average of 2.5 fold. Two populations were examined in more detail. The activity of the secreted cellulase mixtures increased more than two-fold relative to the ancestor and the largest increase was in the extracellular β-glucosidase activity. qPCR showed at least 16-fold more transcribed RNA for *egl4* (endoglucanase IV gene), *cbh1* (cellobiohydrolase I gene) and *bgl1* (extracellular β-glucosidase I gene) in selected populations as compared to the ancestor, and earlier peak expressions of these genes. Deep sequencing shows that the regulatory strategies used to alter cellulase secretion differ in the two strains. The improvements in cellulose deconstruction come from earlier expression of all cellulases and increased relative amount of β-glucosidase, but with small increases in the total secreted protein and therefore little increase in metabolic cost.

## Introduction

Humans have selected fungal strains to improve fermentations for over a thousand years [1]. The earliest selections occurred without an understanding of natural selection, genetic inheritance and the molecular basis of living systems. Today’s better understanding of proteins often permits engineering of improved variants [2], but it remains difficult to engineer improvements in complex biosynthetic pathways [3] or complex traits [4].

The fungus *Trichoderma citrinoviride* (teleomorph *Hypocrea schweinitzii*, Ascomycota), is a widespread soil fungus of the longibrachiatum clade in the genus *Trichoderma* [5]. Its genomic DNA shows 82% nucleotide sequence identity with the more widely studied *T*. *reesei*. It deconstructs cellulose by secreting mixtures of cellulases: endocellulases, cellobiohydrolases and β-glucosidase (S1 Fig). Rational engineering of *Trichoderma* spp. for faster deconstruction is difficult because *Trichoderma* spp. secretes more than ten cellulases [6], which cooperate to yield more glucose than the sum of the separately acting cellulases [7]. Other complications include the possible contribution of 1) oxidases that cleave of cellulose chains [8], 2) enzymes that disrupt the crystallinity of cellulose [9] and 3) xylan-degrading enzymes.

Experimental evolution of microbes has previously improved their growth in different environments [10]. Improvement occurs by variation due to errors in DNA replication followed by selection [11]. One does not need to know which molecular changes are needed. Our goals were first, selection of populations of *Trichoderma* that deconstruct cellulose more efficiently and second, molecular insight into the changes that caused the increased efficiency.

Another potential advantage of experimental evolution is strain stability. For example, one way to increase cellulose deconstruction would be to increase the amount of secreted cellulases. Although such variants would deconstruct more cellulose, they would also grow more slowly since a larger fraction of their metabolic resources are used to make cellulases [12]. If a mutation occurs to reduce the amount of secreted cellulases, the mutant will grow faster and eventually dominate the population [13,14]. Thus, this engineered strain is less stable than the wild type strain because selection pressure favors loss of this engineered improvement. In contrast, the experimental evolution approach selects for stable solutions [15]. Indeed, the populations described below secrete similar total amounts of cellulase, but earlier and in more efficient combinations. Secreting more cellulase would create an additional fitness cost, but secreting different cellulase when the total amount remains similar does not impose a protein synthesis cost.

Experimental evolution for growth on an insoluble substrate like cellulose is more difficult than for growth on a soluble substrate. Growth on an insoluble substrate requires secreted extracellular enzymes, but all microbes in the population benefit from these enzymes. Microbes that do not produce enzymes (cheaters) can grow faster that those that do produce enzymes. Eventually no enzyme producers remain, which leads to extinction of the population [16].

Our design of the experimental evolution created times and places where improved microbes would benefit from their improvements [17]. *T*. *citrinoviride* populations were propagated in serial culture conditions with filter paper as the sole source of carbon. Serial transfer to fresh media selects for rapid growth [18] and creates a time with low exoenzyme concentrations. The low exoenzyme concentrations favor the exoenzyme-producing variants because they benefit from the enzymatic activity immediately, while the cheaters must wait for the exoenzymes to accumulate throughout the medium. We transferred paper strips, not culture liquid, to fresh media to select for fungi that attach to paper strips. This attachment localizes enzymatic action and release of cellulose breakdown products to the region surrounding the exoenzyme-producing variants where they can benefit. Cheating variants in solution or on other regions of the cellulose benefit less from the exoenzymes. These experimental details were critical to the success of the experiments below, but may also limit the size of the improvements that can be achieved.

## Materials and Methods

### General

*Trichoderma viride* strain FP-102208 was obtained from Forest Products Lab, WI. DNA sequencing of the internal transcribed spacer (ITS, primers are listed in Table 1) identified this strain as *Trichoderma citrinoviride [19]*. Protein concentrations were measured by the Bradford dye-binding assay at 595 nm using the Bio-Rad reagent and five dilutions of bovine serum albumin as a standard. Protein gels were run on sodium dodecyl sulfate polyacrylamide gradient gels (NuPAGE^®^ 4–12% Bis-Tris Gel from Invitrogen, MOPS-SDS buffer), using BenchMark protein ladder (5 μL/lane) as the standard. The gel was stained using SimplyBlue^™^ SafeStain (Invitrogen). DNA was sequenced by ACGT, Inc (Wheeling, IL).

**Table 1:** Oligonucleotides for amplifying the internal transcribed spacer (ITS) region, amplifying the cellulase genes *cbh1*, *cbh2* and *bgl1*, and for qPCR of various *T*. *citrinoviride* genes.

Name	Sequence
ITS Forward	5’ TCC GTA GGT GAA CCT GCG G 3’
ITS Reverse	5’ TCC TCC GCT TAT TGA TAT GC 3’
*cbh1* Forward	5’ CAT GTA TCG GAA GTT GGC CGT C 3’
*cbh1* Reverse	5’ GCT TTA CAG GCA CTG AGA GTA GTA AGG 3’
*cbh2* Forward	5’ CAT GAT TGT CGG CAT TCT CAC CAC 3’
*cbh2* Reverse	5’ CCT TAC AGG AAC GAT GGG TTT GC 3’
*bgl1* Forward	5’ GAA ATG CGT TAC CGA ACA GCA GC 3’
*bgl1* Reverse	5’ CTC GCT CCC ACG CTG ATG 3’
*egl4* Forward (qPCR)	5’ GTC ACA GGC ACT GGA GAC C 3’
*egl4* Reverse (qPCR)	5’ GAT CCG TCG CCT GGT AAA G 3’
*cbh1* Forward (qPCR)	5’ GCT ACG ACG GCA ATA CTT GG 3’
*cbh1* Reverse (qPCR)	5’ GTT CTT CGC GCA AGT CTC AT 3’
*bgl1* Forward (qPCR)	5’ CAG GCT ATC CGT CGT TCA A 3’
*bgl1* Reverse (qPCR)	5’ TGA CGT TGG TCT TGT GGT TC 3’
*sar1* Forward (qPCR)	5’ CTC TCC AAG GTT CCC TTC GT 3’
*sar1* Reverse (qPCR)	5’ CAG CTC GTC CTC GGA AAC 3’
*act* Forward (qPCR)	5’ GCT GAG CGT GGT TAC ACC TT 3’
*act* Reverse (qPCR)	5’ CTT GAT GTC ACG GAC GAT TTC 3’

### Experimental evolution

Ten replicate populations of *T*. *citrinoviride* were grown in test tubes (25 × 150 mm) containing 10 mL of minimal filter paper media at 28°C and 240 rpm. Minimal filter paper media contained filter paper strips (VWR^®^ Grade 415, 0.10 g, ~30 strips measuring 1 × 15 mm) as the carbon source suspended in 10 mL of mineral salt solution ((NH_4_)_2_SO_4_ 40 mg, KH_2_PO_4_ 100 mg, MgSO_4_•7H_2_O 25 mg, CaCl_2_ 5 mg, MnSO_4_•H_2_O 0.05 mg, CuSO_4_•5H_2_O 0.01 mg, FeSO_4_•7H_2_O 0.1 mg, ZnSO_4_ 0.1 mg per liter). For the first 45 transfers, ~8 paper strips were transferred to fresh media every 3 days. After the 45^th^ transfer, the paper strips were transferred to fresh media every 2 days. For further characterization, the populations at the 45^th^, 85^th^ and 125^th^ transfers were grown for 7 days to produce conidia and stored frozen at -80°C in glycerol stocks (0.8 mL culture and 0.2 mL glycerol).

### Amount of filter paper deconstructed

Minimal filter paper media (10 mL, ~100 mg paper) in a test tube was inoculated with conidia (~10^6^) from ancestral and selected (at 45^th^, 85^th^ and 125^th^ transfer) *T*. *citrinoviride* populations and incubated at 28°C and 240 rpm for 5 days. The populations were filtered through porcelain Büchner funnel without filter paper to collect the remaining filter paper. This filter paper was transferred to glass vial, dried in an oven (100°C) overnight and weighed. A few mycelia may stick to the paper, but the amount is insignificant compared to the amount of paper. The dry biomass of *T*. *citrinoviride* is 5–6% of the wet biomass, so the expected amount of dry mycelia from S3 Fig is 0.25–0.75 mg, which is <1% of the amount of filter paper (100 mg).

### Amount of mycelia

The amount of mycelia was measured by extracting ergosterol from the mycelia and measuring the amount by HPLC [20]. Fungal mycelia were harvested, frozen in liquid nitrogen for 0.5 h, ethanol (4 mL) was added to the frozen sample and then it shaken at room temperature for 2 h. An aliquot of KOH solution (60% v/w, 0.8 mL) was added to the mixture, which was then heated to 97°C for 20 min. This sample was cooled and neutralized with HCl (36.5%, ~0.75 mL). The solution was extracted with hexane (2 x 5 mL), the hexane fractions were combined and evaporated by rotary evaporation. The residue was dissolved in ethanol (1 mL), filtered through a 0.22 μm membrane filter and analyzed by HPLC on a reversed phase column (ZORBAX Eclipse Plus C18, 4.6 x 250 mm, 5 μm particle size, Agilent) eluted a 1.0 mL/min with methanol-water (97:3 v/v) with the UV detector set to 280 nm. A standard curve prepared with known amounts of *T*. *citrinovirde* wet biomass (corresponds to 5–6% dry weight after drying overnight at 100°C) was used to convert the measured amounts of ergosterol to amount of biomass.

### Isolation of secreted proteins

Carboxymethyl cellulose media (50 mL) in a 250-mL Erlenmeyer flask was inoculated with conidia (~5 × 10^6^) from ancestral and selected (at the 125^th^ transfer) *T*. *citrinoviride* populations. Carboxymethyl cellulose media contained sodium carboxymethyl cellulose (CMC, average MW ~90000, Sigma) 10 g, yeast extract (BD Bacto^™^) 0.25 g, peptone (Fisher) 0.75 g, urea 0.30 g, (NH_4_)_2_SO_4_ 1.4 g, KH_2_PO_4_ 2.0 g, CaCl_2_ 0.30 g, and MgSO_4_ 0.30 g in 1 L water. The populations were incubated at 28°C, 120 rpm for 2–7 days. The mycelium was removed by filtration with suction through a bed of glass wool, the filtrate was chilled in an ice bath for 30 min, then centrifuged at 13000 rpm for 20 min to remove any remaining particles. The supernatant was transferred to a beaker, solid ammonium sulfate was slowly added with stirring to 80% saturation at 4°C and kept at 4°C for 5 h. The precipitated proteins were collected by centrifugation (13000 rpm, 30 min), dissolved in citrate buffer (15 mL, 50 mM, pH 5.0) and concentrated to 2 mL by centrifugal ultrafiltration using a 10 K molecular weight cut off membrane. The solution was stored at 4°C and analyzed as described below.

### Cellulase activity assays

Cellulase activity assays for filter paper (FP activity), carboxymethyl cellulose (CMC activity) and Avicel were performed as literature [21], and the released reducing sugars were measured by DNS assay, 0.4 mL sample and 0.6 mL DNS assay solution were combined in a microcentrifuge tube (1.5 mL), heated at 95°C for 3 min, and then cooled in ice-water bath for 5 min. The absorbance at 540 nm was measured and compared to a calibration curve prepared using glucose solutions (0.6–30 mg/L) [22]. One unit of activity corresponds to the release of 1 μmol of reducing sugars as glucose equivalents per min. β-Glucosidase activity was measured with 4-nitrophenyl β-D-glucopyranoside (pNPG) as the substrate [23]. One unit of β-glucosidase activity corresponds to the release of 1 μmol of *p*-nitrophenol per min.

### Protein identification by mass spectrometry of trypsin-digested fragments

The proteins were separated by sodium dodecyl sulfate polyacrylamide gel electrophoresis and the gel regions containing the strong bands at ~50, 55, and 72 kDa were cut out, and treated with trypsin in-gel [24]. Mass spectrometry and protein database searching on tryptic peptides [25] used the database nr_Trichod_reesei 51453_20130403_cRAP123 with 9761 protein entries.

### Cellobiohydrolase I and II and β-glucosidase I gene sequencing

The genomic DNA of ancestral and selected (F1 and F4) *T*. *citrinoviride* were isolated by DNeasy^®^ Plant Mini Kit (Qiagen). The genes *cbh1*, *cbh2* and *bgl1* were amplified using primers in Table 1 and using the isolated genomic DNA as the template. These primers were designed based on the ends of the corresponding sequences from *T*. *viride*, except for the reverse primer for *bgl1*. It was designed based on a conserved region within the gene 90 bases from the 3’ end. The amplified genes were purified by agarose gel electrophoresis and cloned into pGEM^®^-T vector (Promega) for sequencing. The plasmid was transferred into competent *E*. *coli* DH5α, which was plated on X-Gal/IPTG LB-ampicillin (50 μg/mL) agar plate for blue-white screen. A white colony was picked and used to inoculate LB-ampicillin (50 μg/mL) media (3 mL), after growth overnight, the plasmid DNA was isolated and sequenced by primers based on T7 and SP6 promoter.

### RNA extraction and expression profiling by qPCR

Carboxymethyl cellulose media (CMC, 50 mL) in a 250 mL Erlenmeyer flask was inoculated with conidia (~ 5 × 10^6^) from ancestral and selected (F1 and F4 at the 125^th^ transfer) *T*. *citrinoviride* populations. The cultures were transferred to tubes at 16, 20, 24, 28, 36 and 48 h and centrifuged (13000 rpm, 4°C, 20 min) and the supernatants were discarded. The harvested hyphae were resuspended in RNAlater solution (Sigma, 3 mL), and then RNA was purified using the RNeasy Plant Mini Kit (Qiagen) following the manufacturer’s instructions. The RNA was transcribed to cDNA using SuperScript II RT from Invitrogen. qPCR was performed on the ABI 7900 HT. 3 μl of cDNA (15 ng) and 3 μl of mix (10 μM forward primer (Table 1), 10 μM reverse primer (Table 1), 10 μM probe (Roche Universal Probe Library), and 2.22 X Home brew master mix) were dispensed into duplicate wells on a 384-well ABI optical plate. The plate was inserted into the ABI 7900 HT. The FAM-Non Fluorescent Quencher detector and Rox passive reference was selected. The qPCR protocol was initiated: 2 min activation at 60°C and a 5 min denaturation at 95°C, followed by 45 cycles of 10 s at 95°C and 1 min at 60°C. The expression levels of *sar1* and *act*, which are housekeeping genes in *T*. reesei [26], were used to normalize the data from the different samples [27].

### RNA sequencing

Mycelia from ancestral and selected *T*. *citrinoviride* grown for 2 days in CMC media were harvested and immediately submerged in RNAlater and RNA were purified using the RNeasy Plant Mini Kit (Qiagen) following the manufacturer’s instructions. Total RNA was used as template for the construction of a cDNA library for next-generation sequencsing using paired end reads with lengths of 101 base pairs on the Illumina HiSeq 2000 by U. of Minnesota Genomics center. Each RNA-seq library consisted in over 14 M reads of the 101-bp paired-end sequence, which resulted ~100 x coverage of *T*. *citrinoviride* transcriptome (1481 bp average transcript size x 9737 loci = 14.4 Mbp; data from Trici4, Joint Genome Institute, http://genome.jgi.doe.gov/Trici4/Trici4.home.html). Assembly and analysis of the RNA-seq library was performed in Galaxy (http://usegalaxy.org). Sequences were assembled based on the genome of *T*. *citrinoviride* v4 (33.22 Mbp) using Tophat2 (http://ccb.jhu.edu/software/tophat/index.shtml) [28]. Differential expression testing was performed by Cuffdiff (http://cufflinks.cbcb.umd.edu/) [29].

## Results

### Selection of *T*. *citrinoviride* for faster growth on cellulose

Ten replicate populations of *T*. *citrinoviride* (10 mL) were grown on filter paper strips (0.1 g, approximately 30) suspended in mineral media. After 3 days, some of filter paper broke up into fibers and the biomass reached ~5 mg wet cell weight (~0.25 mg dry cell weight), and the growth neared the end of the exponential phase. About 8 partly deconstructed strips of filter paper were transferred to fresh media, S2 Fig. For the first 45 transfers, the populations were transferred every three days. Over the next 80 transfers, the filter paper broke up more quickly, so they were transferred every two days. None of the cultures went extinct during these transfers. The selected populations after 125 transfers yielded up to 3 times more hyphal biomass than the ancestor (S3 Fig).

The filter paper inoculum contained <0.2 mg wet cell weight of biomass, so the population dilution is 5/<0.2 = >20-fold which corresponds to >4.3 generations per transfer (log_2_(20) = 4.3). An alternative inoculum, transfer of liquid (0.1 mL, with paper fibers), did not yield stable populations. About half of the ten populations went extinct after four liquid transfers and the filter paper remained intact after three days. Biomass measurements based on ergosterol amounts showed that 90% of the hyphae stuck to the filter paper, so transferring paper was the most effective way to transfer biomass.

### Increased filter paper deconstruction

**Fig 1:**
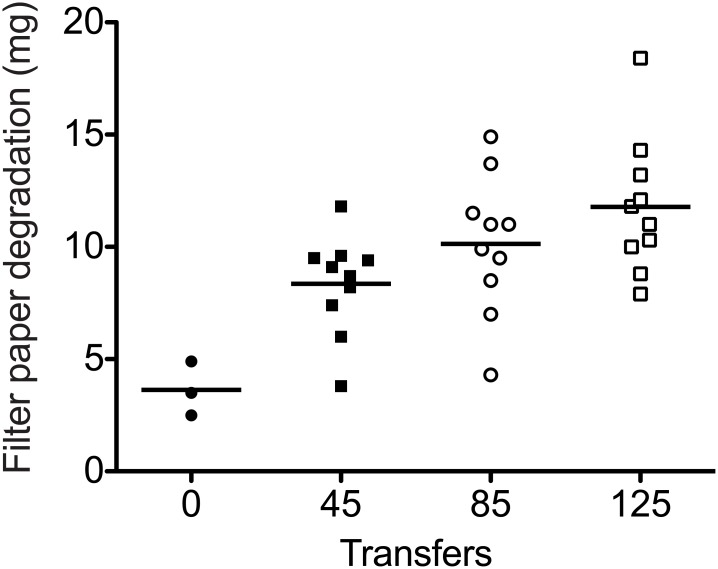
The selected (45^th^, 85^th^ and 125^th^ transfer) populations of *T*. *citrinoviride* deconstructed more filter paper than the ancestral population. The horizontal lines indicate the average of each cluster and the error bars indicate the standard deviation. Filter paper deconstruction increased greatly during the first 45 transfers; then more slowly during additional transfers.

After 125 transfers (~540 generations), all ten *T*. *citrinoviride* populations deconstructed 2.3 to 5.1 times more filter paper than the ancestral population (P < 0.0001, ANOVA planned comparison), Fig 1. To measure the deconstruction for each replicate population, filter strip media was inoculated with ~10^6^ *T*. *citrinoviride* conidia. After 5 days growth, the remaining paper (80–95 mg) was collected by filtration through a Büchner funnel (~0.7 mm holes), dried at 95°C overnight and weighed. The contribution of adhering fungal hyphae was <1% of the amount of filter paper. The paper contained 5–15 mg of wet cell weight (S3 Fig), which corresponds to ~0.25–0.75 mg after drying. Less filter paper remained in the selected populations than in the ancestor populations confirming that the selected populations grow faster. After the first 45 transfers, the ten selected populations deconstructed an average of 2.3-fold more filter paper than the ancestor (P = 0.0001, ANOVA planned comparison). All selected populations showed substantial improvements. Thus, all the *T*. *citrinoviride* populations adapted quickly during these first 45 transfers to deconstruct filter paper more rapidly. During the next forty transfers (85 total transfers), the average amount of filter paper deconstructed increased slightly to 2.8-fold over ancestor (P < 0.0001, ANOVA planned comparison). These forty transfers yielded a smaller increase (1.2-fold on average) in the amount of filter paper deconstruction as compared to the first 45 transfers (P 0.0213, ANOVA planned comparison). During the next forty transfers (125 total transfers), there is a small further increase in the amount of paper deconstructed to an average of 3.2-fold faster than ancestor, but this increase was not statistically significant over the amount at 85 transfers (P 0.1072, ANOVA planned comparison).

### Increased protein expression and catalytic activity

Growth on an insoluble substrate like filter paper requires microorganisms to secrete enzymes that break the substrate into fragments that can transported into the cell. To measure these secreted proteins we grew the *T*. *citrinoviride* on a soluble cellulose derivative, carboxymethylcellullose (CMC) [30]. The use of a soluble substrate avoids possible absorption of the proteins on the insoluble substrate.

**Fig 2:**
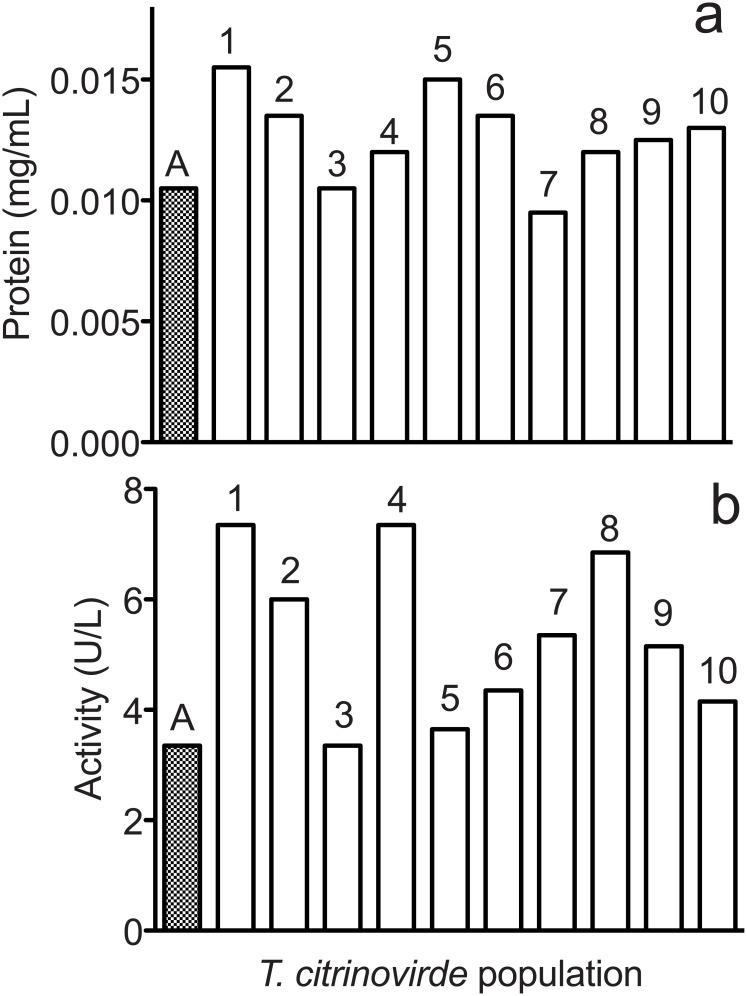
Extracellular protein amounts (panel a) in ancestral and selected *T*. *citrinoviride* populations are similar (range 0.9–1.5-fold of the ancestor), but the extracellular specific activity (panel b) is at least two fold higher in four of the ten selected populations. The symbol indicates the ancestor and the numbers indicate the selected populations that named as “F” and the number. Conditions: 50 mL media containing 10 g/L CMC inoculated with ~ 5 × 10^6^ conidia, 5 d growth.

The total amounts of secreted protein were similar for both ancestor and selected populations after 5 days (range 0.9 to 1.5-fold of the ancestor, *P* = 0.1176, ANOVA planned comparison), Fig 2a. Populations F1, F2, F5, F6, F9 and F10 secreted 1.2–1.5 times more protein than ancestor, populations F3, F4, F7 and F8 secreted similar amounts (0.9–1.1-fold). Since both ancestor and selected populations secreted similar total amount of protein, but the fraction of cellulases in this total was unknown, the faster paper-deconstructing (Fig 1) abilities of the selected populations could come from 1) more efficient filter-paper-deconstructing enzymes, 2) a higher fraction of filter-paper-deconstructing enzymes in the total secreted proteins, 3) earlier secretion of the filter-paper-deconstructing enzymes, so they are present for a longer time, or 4) a more effective mixture of enzymes.

The cellulase activity in terms of filter paper units (FPU, per unit of culture volume) was higher for all but one of the selected populations as compared to the ancestor (range = 1.0 to 2.5-fold higher, *P* = 0.0017, ANOVA planned comparison), Fig 2b. Filter paper deconstruction activity was measured by the dinitrosalicylate assay, which measures the release of reducing sugar. The average increase was 1.6-fold over the ancestor. Populations F1 (7.35 U/L, 0.47 FPU/mg protein) and F4 (7.35 U/L, 0.62 FPU/mg protein), which showed the highest activity for filter paper hydrolysis (2.5-fold higher than ancestor (3.05 U/L, 0.32 FPU/mg protein)), were further characterized.

### Secreted proteins and enzyme activity of F1 and F4

**Fig 3:**
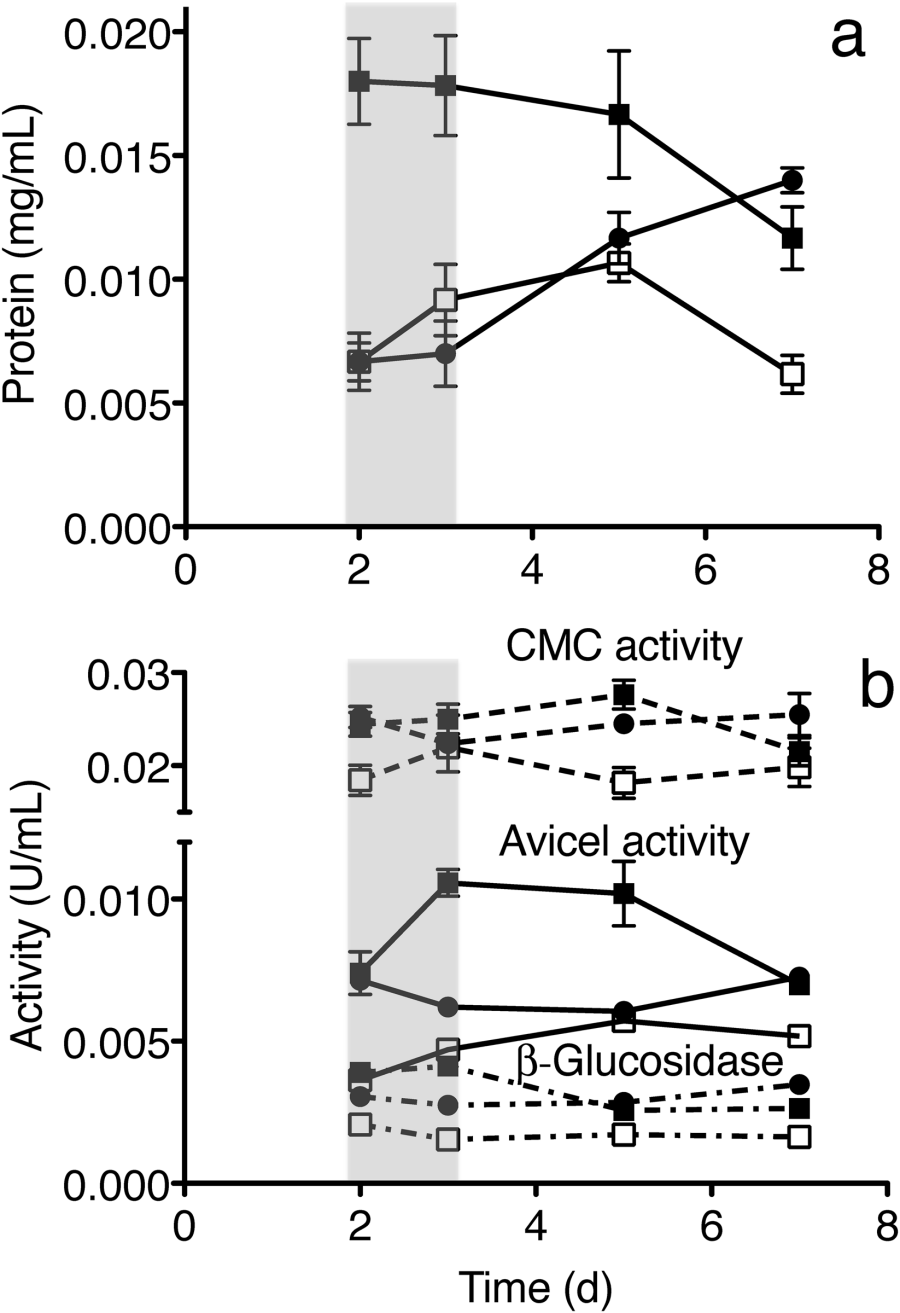
Amounts of secreted protein and specific activity in culture for several populations. a) The amount of extracellular protein during days 2–5 in population F1 (closed square, 125 transfers) was about twice the amount in population F4 (closed circle, 125 transfers) and in the ancestral (open square) *T*. *citrinoviride*. During selection, the populations were transferred at 2–3 days (gray shading), so changes after this time do not influence adaptation. b) The specific activity of the secreted proteins remained constant over days 2–7 for both populations F1 (closed square, 125 transfers), F4 (closed circle, 125 transfers) and the ancestral population (open square): CMC activities (dashed line, estimates endocelluase activity), Avicel activities (solid line, estimates cellobiohydrolase activity) and β-glucosidase activities (dot-dash line). The populations F1 and F4 showed similar CMC activity as the ancestor, but approximately two-fold higher of Avicel activity and three-fold higher β-glucosidase activities. Conditions: CMC-media was inoculated with the same number of conidia from each strain and cultured at 28°C. The secreted proteins were harvested at days 2, 3, 5 and 7, and assayed for activity. See Supporting Information for details.

Population F1 secreted twice as much protein as the ancestor after 2 days growth in CMC media, while population F4 secreted similar amounts of protein as the ancestor, Fig 3a. The secreted protein concentration in population F4 continued to increase at days 5 and 7, but this increase does not directly reflect an adaptive benefit for the population, because the populations were transferred at days 2 or 3. The decrease protein concentration of ancestor and F1 at day 7 may be from the protein degradation or aggregation.

The specific activity of population F1 and F4 was two-fold higher in the Avicel assay (mainly cellobiohydrolase activity) compared to the ancestor and ~3-fold higher in the β-glucosidase assay, but similar (1.2-fold higher) in the CMC assay (mainly endocellulase activity), Fig 3b. Thus, the largest increase was at the last step of the cellulose deconstruction, a three-fold increase in β-glucosidase activity for hydrolyzing cellobiose into glucose. Since the total amount of protein increased less than three fold, the activity of the enzymes may have increased or the relative amount of the enzymes increased as a fraction of the total.

### Changes in cellulase secretion

SDS-PAGE analysis of secreted proteins showed three main bands at about 50, 55 and 72 kDa. The selected populations secrete slightly more of the proteins at 50 and 55 kDa and twice as much of the protein at 72 kDa (S4 Fig). Protein sequencing by LC-MS/MS identified the band with a mass of 50 kDa as cellobiohydrolase II (CBHII) and six other glycoside hydrolases belonging to glycoside hydrolases family 5, 15, 30 and 54 (S1 Table). Population F1 and F4 secreted 0.8-fold and 1.6-fold more of the proteins in this band. The 55-kDa band was identified as cellobiohydrolase I (CBHI) and a glycoside hydrolase family 5 protein. Population F1 and F4 secreted 1.3-fold and 1.4-fold of the proteins in this band. The 72-kDa band was identified as β-glucosidase I (BGLI) and two glycoside hydrolase family 55 proteins. The relative amount of the 72-kDa band increased two-fold for both populations F1 and F4, from 6% of the total protein to 12%, Table 2. The less prominent bands in the SDS-PAGE, which appear at 24, 30, 38, 43 and 48 kDa are endoglucanases of *Trichoderma* spp. [6]. The gels showed no major changes in these bands (S4 Fig).

**Table 2:** The relative amounts of secreted proteins in the major molecular weight bands as determined by SDS-PAGE.^a^

Band (kDa)	Ancestor	F1	F4
50	17%	13%	28%
55	10%	13%	14%
72	6%	12%	12%

^a^The amounts were measured by band intensity on SDS-PAGE using ImageJ. The amounts do not add up to 100% because the SDS-PAGE gel also contains other, uncharacterized, secreted proteins at other molecular weights (S4 Fig).

### Unchanged cellobiohydrolases I & II and β-glucosidase I genes

The DNA sequences encoding cellobiohydrolases (*cbh1* and *cbh2*) and β-glucosidase I (*bgl1*) are identical in the selected populations (F1 and F4) and in the ancestral genotype. The *cbh1*, *cbh*2 and *bgl1* were amplified by polymerase chain reaction from the ancestral and selected *T*. *citrinoviride* genomes. Sequencing showed no differences between them, so changes in the relative amounts and earlier secretion (see below) account for the faster cellulose deconstruction of the selected populations.

### Increased transcription of cellulose-deconstruction genes

**Fig 4:**
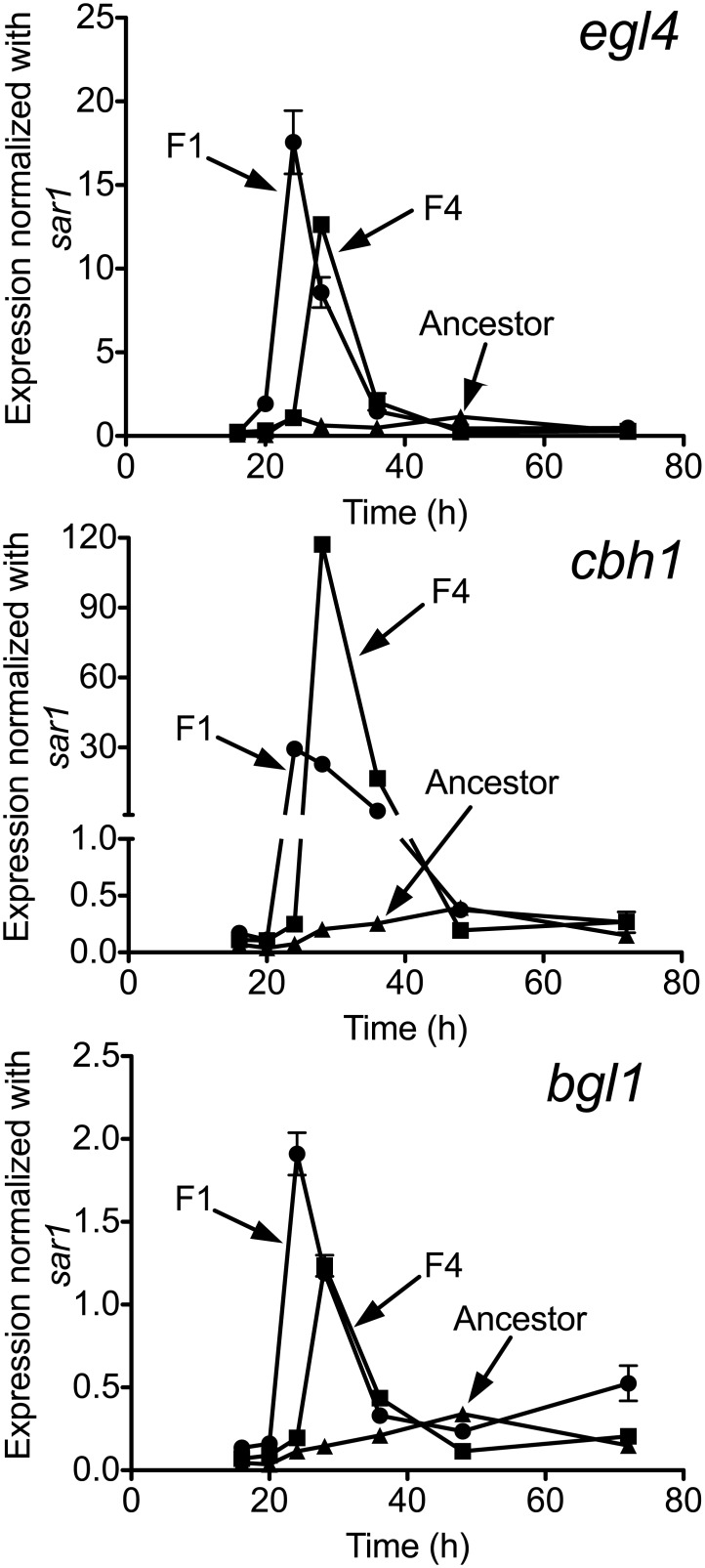
RNA transcriptions of *egl4*, *cbh1* and *bgl1* genes at 24 h are up to 500-fold higher in populations F1 (circle) and F4 (square) than in the ancestor (triangle). Culture of CMC media (40 mL) were inoculated by conidia (~ 5 × 10^6^) and RNA was extracted from hyphae collected at 16, 20, 24, 28, 36, 48 and 72 h and measured by qPCR. The error bar means standard error of the mean.

When grown on filter paper most of the mycelia cling to the paper. To isolate the mycelia for measuring RNA transcript levels, we used a soluble carbon source, CMC. This media was also used above to measure amounts of secreted cellulases. The mRNA transcript levels measured by qPCR, show that populations F1 and F4 transcribe up to 500-fold more copies of the *bgl1*, *cbh1* and *egl4* genes than the ancestor (Fig 4, S2, S3 and S4 Tables). Population F1 has the highest cellulase gene transcription at 24 h, F4 has the highest at 28 h, but the ancestor has the highest at 48 h. *egl4* transcription in the ancestor also has a second peak at 24 h. At their peak transcription times, populations F1 and F4 transcribe about 15-fold, 500-fold and 5-fold more of the *egl4*, *cbh1* and *bgl1* genes than the ancestor, respectively. The selected populations have large, early transcription peak for these cellulases, but the ancestor has lower and relatively constant transcription levels. The expression of other glycoside hydrolases—there are at least 10 more glycoside hydrolases identified in the related *T*. *reesei* [31]—were not measured. The large early peak transcription peaks in the selected populations leads to more secreted cellulases and faster deconstruction of filter paper.

Next generation sequencing of RNA from both selected and ancestral populations of *T*. *citrinoviride* identified different regulatory changes that increased the secretions of cellulases in the two populations (Table 3). We focus on those transcription regulators that were previously shown to affect cellulase secretion in *T*. *reesei*. The carbon catabolite repressor genes ACE1, CRE1 and CRE2, which repress transcription in *T*. *reesei* [32,33], have similar transcription levels (within 25%) in ancestral *T*. *citrinoviride* and selected populations F1 and F4. Of the twelve activators of transcription identified in *T*. *reesei* [33–35], eight show similar transcription levels (within 50%) in ancestral *T*. *citrinoviride* and selected populations F1 and F4 (Table 3). In population F1, BglR, which up-regulates expression of β-glucosidases [36], and VEL1, which impairs the expression of cellulases in the *vel1* knockout *T*. *reesei*, are almost two-fold higher [37]. In contrast, in population F4 the expression of these genes is similar, but the expression of two other regulatory genes has increased. GRD1 [38] and LAE1 [39], which enhance cellulase gene expression in *T*. *reesei*. are 4.7- and 1.8-fold higher, respectively, in population F4. Thus, the evolutionary approach identified two different regulation strategies to enhance cellulase secretion.

**Table 3:** The transcription levels of positive and negative regulators of expression of cellulase in *T*. *citrinoviride*.

Positive Regulators			
Gene Name	Trici4 ID	F1/ancestor	F4/ancestor
*ace2*	1121072	0.81	1.06
*hap2*	1121981	1.03	1.25
*hap3*	1121906	1.45	0.69
*hap5*	163357	1.17	0.73
*xyr1*	1171607	0.80	0.44
*clr1*	1169878	1.33	0.60
*clr2*	1181159	1.00	1.12
*bglr*	1162268	**1.73**	0.79
*grd1*	1161214	1.00	**4.70**
*lae1*	1113086	0.94	**1.85**
*vel1*	22542	**1.81**	0.76
*ace3*	1132806	0.63	1.18
Negative regulators			
Gene Name	Trici4 ID	F1/ancestor	F4/ancestor
*ace1*	1118385	1.00	0.97
*cre1*	1109073	0.92	1.25
*cre2*	46888	1.22	0.81

## Discussion

To optimize the cellulose degradation using experimental evolution, we selected replicate populations of the fungi *T*. *citrinoviride* for ~540 generations in media where cellulose breakdown limits growth. Over the course of selection, natural errors in DNA replication (~10^8^ cells in the cultures and 10^−9^ errors per site per cell division [40]) created several billion (~2 x 10^9^) changes. Selection for improved variants resulted in populations with up to five-fold faster deconstruction of cellulose. The amino acid sequence of the cellulases did not change indicating that evolution did not create better enzymes; instead evolution changed the expression of the cellulases. Both the DNA and RNA sequencing are of multiple molecules that reflect the evolved population. The isolation of DNA and RNA collects many individuals and the sequencing shows the results of many individual molecules. Heterogeneity in the sequence would yield a noisy signal, but the signals were strong and clear indicating the population was an effectively pure culture.

**Fig 5:**
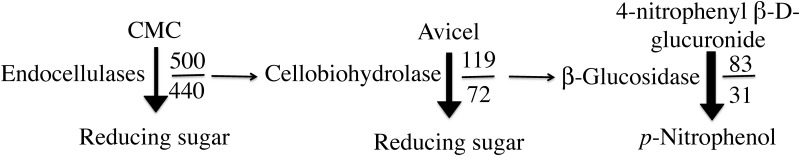
Population F1 showed higher activity (in U/L culture) for all three of the activities needed for cellulose deconstruction as compared to the ancestor (compare top versus bottom numbers). The three types of activities were measured using three different substrates: carboxymethylcellulose (CMC) for endocellulase activity, crystalline cellulose (Avicel) for cellobiohydrolase activity and 4-nitrophenyl β-D-glucopyranoside for β-glucosidase activity. The endocellulase activity increased 1.2-fold in population F1 as compared to the ancestor, the cellobiohydrolase activity increased 2.1-fold and the β-glucosidase activity increased 2.7-fold.

The selected populations degraded filter paper faster because they secreted cellulases earlier, secreted an optimized mixtures of cellulase and secreted slightly more cellulases (Fig 5). Focusing on population F1 after 125 transfers, filter paper deconstruction increased 2.5-fold. The activity increase at each successive step of cellulose hydrolysis was larger. The first, endocellulase, step increased 1.2-fold; the second, cellobiohydrolase, step increased 2.1-fold and the third, β-glucosidase I, step increased 2.7-fold. This pattern of increased activity suggests that the β-glucosidase activity limits cellulose deconstruction in the ancestor. Indeed, degradation of pretreated biomass by *Trichoderma* spp. cellulase mixtures is limited by β-glucosidase causing the accumulation of cellobiose that inhibits endocellulases and exocellulases [21]. Our experimental evolution approach is the only approach that so far has improved *Trichoderma* spp. to secrete an optimal cellulase mixture for cellulose degradation. The specific activity of the cellulase mixture produced by selected populations F1 and F4 (0.47 and 0.62 FPU/mg protein) was higher that the ancestor (0.32 FPU/mg). UV and chemical mutagenesis created variants of *T*. *reesei* RUTC30 that secrete more cellulases, but the specific activity of this cellulase mixture is unchanged because the proportions of the cellulases remained the same [41]. Thus, current industrial strains produce more cellulases, but our evolved strain produces a better mixture.

Although some mRNA transcripts increased 500-fold, the amounts of the corresponding proteins increased only about two-fold. RNA transcripts for *egl4*, *cbh1* and *bgl1* increased 5- to 500-fold in the selected populations relative to the ancestral genotype. In contrast, the amount of extracellular protein encoded by this RNA increased at most only approximately two-fold. Although the increase mRNA transcripts for *egl4* and *chb1* are larger than those for *bgl1*, the increase in the EGIV and CBHI secretion was smaller than BGLI. The RNA stability and translation step may cause this mismatch between increases in mRNA and protein amounts. The regulatory mechanism for cellulase secretion changes in the selected *T*. *citrinoviride* population F1 and F4 were different, cellulase expression positive regulators *bglr* and *vel1* were up-regulated in population F1, but *grd1* and *lae1* were up-regulated in population F4. This indicate multiple paths to increase the cellulase expression in *T*. *citrinovirde*.

Why aren’t natural *T*. *citrinoviride* already optimized for deconstruction of cellulose? One reason is that natural biomass differs from filter paper. In natural biomass, cellulose is intertwined with lignin and hemicellulose, which hinders access to the cellulose. Large pieces allow access to only the small amounts of cellulose on the surface. The initial cleavage of cellulose is likely the rate-limiting step, requiring large amounts of the first cellulose-deconstructing enzymes. In contrast, cellulose is more accessible in filter paper. Second, natural biomass deconstruction occurs on damp, solid surfaces where diffusion is slow, while our experiments used agitated liquid media. Secreted enzymes stay nearby when diffusion is slow, but diffuse away in stirred media, so different ratios of enzymes may be optimal for the two conditions. Third, nature constantly changes, so natural strains encounter different conditions, different types of biomass as well as competing organisms. Natural strains must also be optimized to thrive in a range of conditions, and secrete secondary metabolites to compete with other microorganims. In contrast, our experiments used a narrow, unvarying growth conditions with no competing microorganisms.

The increase in filter paper degradation activity appears to be reaching a plateau. The average value after 125 transfers was not significantly better than after 85 transfers. The overall improvement of appears to have reached a limit of only five-fold faster cellulose deconstruction. One hypothesis is that selecting for improved exoenzyme activity on the insoluble substrate limited the improvements. The benefit gained by the improved variants must outweigh the cost of producing the enzymes. If the selection conditions are not sufficiently heterogeneous, then the improved variants reap only some of the benefits of improvements. An altered selection strategy might lead to further improvements. Another hypothesis is that filter paper degradation no longer limits growth.

In conclusion, this experimental evolution approach combined with genomic and proteomic analysis of the improved populations is a powerful approach for understanding and optimizing complex phenotypes like cellulose deconstruction.

## Supporting Information

S1 Fig Enzymatic deconstruction of cellulose to glucose. Endocellulases cleave within the chains in the amorphous region of cellulose. Cellobiohydrolases release the disaccharide cellobiose from either the reducing end of the chain (cbhI) or the non-reducing end of the chain (cbhII). β-Glucosidase cleaves cellobiose into two glucose molecules (open white circles).
(PDF)

S2 Fig Selection of *T*. *citrinonviride* for faster growth in minimal filter paper media. Serial transfer of 8 strips of partly degraded filter paper from *T*. *citrinonviride* cultures to fresh media every 2–3 days selected for variants that degrade filter paper faster.
(PDF)

S3 Fig Growth of ancestral and selected populations. All of the ten selected populations (indicated by numbers) accumulated similar or greater amounts of hyphae as compared to the ancestor (Ⓐ) in filter paper media after 3 days.
(PDF)

S4 Fig SDS-PAGE of proteins secreted by ancestor, F1 and F4 populations at days 2 and 3. The amounts of proteins at molecular weights of ~50, 55 and 72 kDa are higher in the selected populations as compared to the ancestor population. These bands were cut from the gel, the proteins were digested with trypsin and the resulting peptides identified by LC-MS/MS analysis. The protein bands 50, 55 and 72 kDa contained cellobiohydrolase II, cellobiohydrolase I and β-glucosidase I, respectively, as well as other glycosyl hydrolases, S1 Table.
(PDF)

S1 Table LC-MS/MS analysis of the protein bands at about 50, 55 and 72 kDa.^a^ (DOCX)

S2 Table Amount of RNA transcript in the ancestral *T*. *citrinoviride* population. Lower Ct values indicate more RNA transcript.
(DOCX)

S3 Table Amount of RNA transcript in the F1 selected population. Lower Ct values indicate more RNA transcript.
(DOCX)

S4 Table Amount of RNA transcript in the F4 selected population. Lower Ct values indicate more RNA transcript.
(DOCX)
