# Supplementary figures and images for: Experimental Evolution of Trichoderma citrinoviride for Faster Deconstruction of Cellulose
Source: PLoS One. 2016 Jan 28;11(1):e0147024. doi: 10.1371/journal.pone.0147024 (PMC4731210; doi:10.1371/journal.pone.0147024)

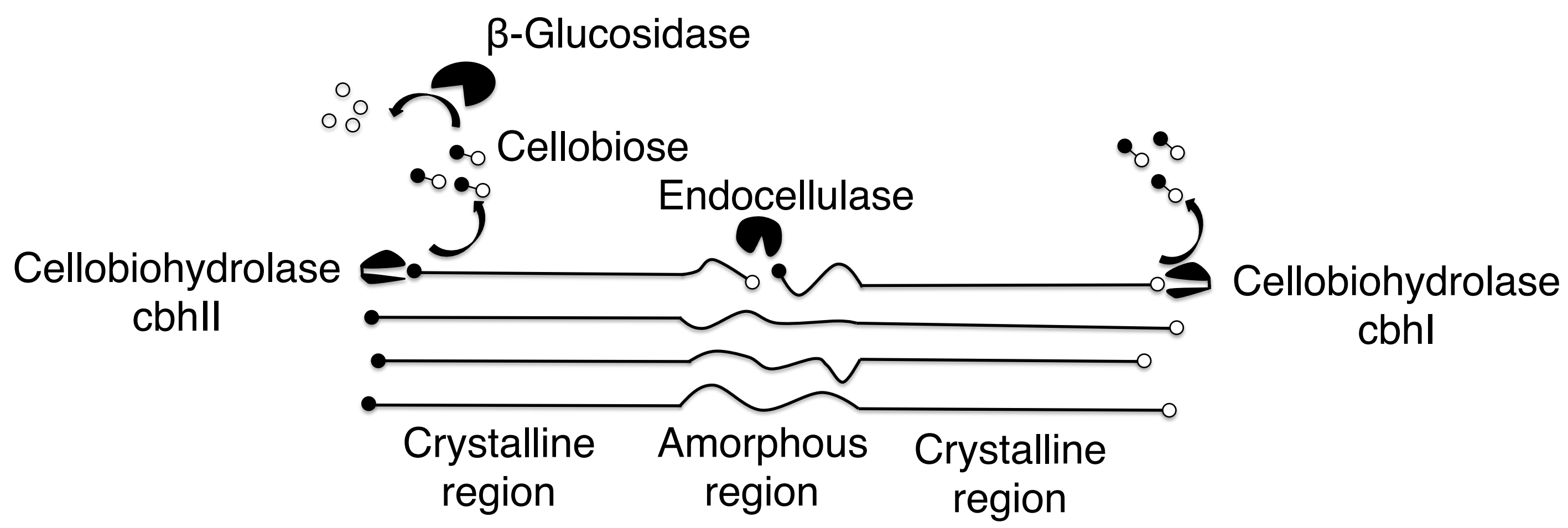

Supplement: S1 Fig — Endocellulases cleave within the chains in the amorphous region of cellulose. Cellobiohydrolases release the disaccharide cellobiose from either the reducing end of the chain (cbhI) or the non-reducing end of the chain (cbhII). β-Glucosidase cleaves cellobiose into two glucose molecules (open white circles). (PDF) [file pone.0147024.s001.pdf]

every 2-3 days

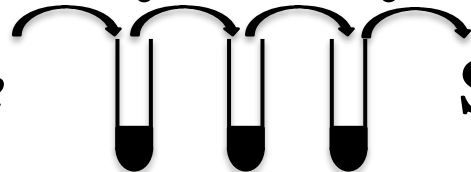

*T. citrinoviride*

Selected populations

Supplement: S2 Fig — Serial transfer of 8 strips of partly degraded filter paper from T. citrinonviride cultures to fresh media every 2–3 days selected for variants that degrade filter paper faster. (PDF) [file pone.0147024.s002.pdf]

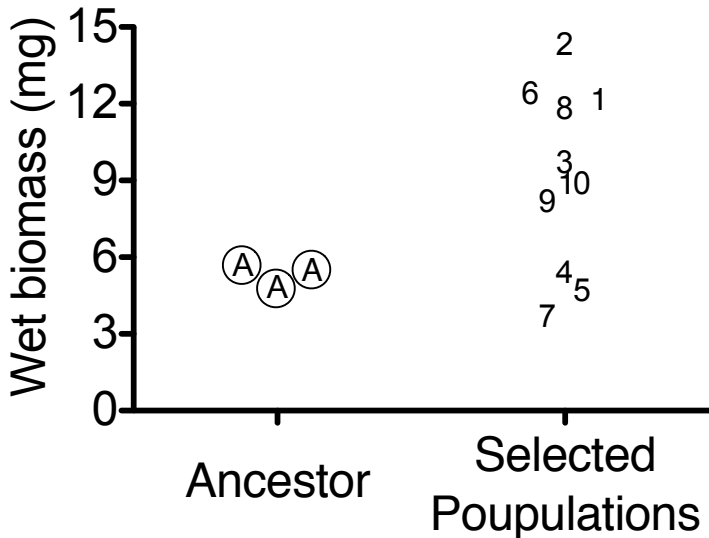

Supplement: S3 Fig — All of the ten selected populations (indicated by numbers) accumulated similar or greater amounts of hyphae as compared to the ancestor (Ⓐ) in filter paper media after 3 days. (PDF) [file pone.0147024.s003.pdf]

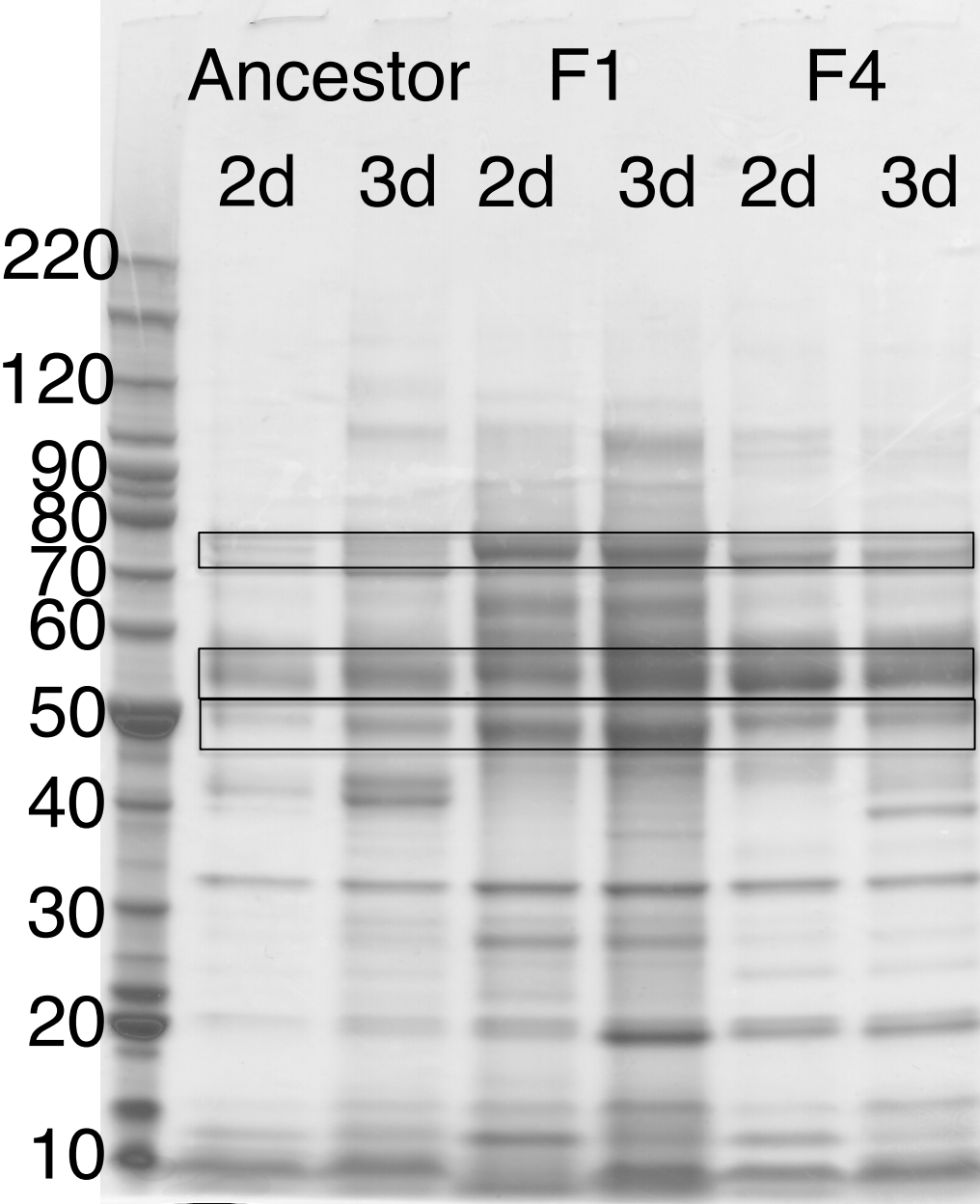

Supplement: S4 Fig — The amounts of proteins at molecular weights of ~50, 55 and 72 kDa are higher in the selected populations as compared to the ancestor population. These bands were cut from the gel, the proteins were digested with trypsin and the resulting peptides identified by LC-MS/MS analysis. The protein bands 50, 55 and 72 kDa contained cellobiohydrolase II, cellobiohydrolase I and β-glucosidase I, respectively, as well as other glycosyl hydrolases, S1 Table. (PDF) [file pone.0147024.s004.pdf]
