# Supplementary material for: Experimental Evolution of Trichoderma citrinoviride for Faster Deconstruction of Cellulose
Source: PLoS One. 2016 Jan 28;11(1):e0147024. doi: 10.1371/journal.pone.0147024 (PMC4731210; doi:10.1371/journal.pone.0147024)
Supplement: S1 Table — (DOCX) [file pone.0147024.s005.docx]

**S1 Table.** LC-MS/MS analysis of the protein bands at about 50, 55 and 72 kDa.*^a^*

| Identified Proteins | Accession Number | Molecular Weight (kDa) | 50 kDa band | 55 kDa band | 72 kDa band |
| --- | --- | --- | --- | --- | --- |
| β-glucosidase I | gi\|227874 | 78 | - | - | + |
| cellobiohydrolase I | gi\|50402144 | 54 | - | + | - |
| cellobiohydrolase II | gi\|121855 | 50 | + | - | - |
| glycoside hydrolase Family 15 | gi\|261825113 | 64 | + | - | - |
| glycoside hydrolase family 30 | gi\|340514491 | 52 | + | - | - |
| glycoside hydrolase family 5 | gi\|340514558 | 53 | + | + | - |
| glycoside hydrolase family 5 | gi\|340515257 | 52 | + | - | - |
| glycoside hydrolase family 5 | gi\|340521228 | 40 | + | - | - |
| glycoside hydrolase family 54 | gi\|340521563 | 53 | + | - | - |
| glycoside hydrolase family 55 | gi\|340518458 | 83 | - | - | + |
| glycoside hydrolase family 55 | gi\|340517276 | 80 | - | - | + |

*^a^*The bands were cut from the gel, digested with trypsin and sequenced by LC-MS/MS. “+” means that protein was detected in the band, and “-“ means that protein was not detected in the band.
