# Supplementary material for: Experimental Evolution of Trichoderma citrinoviride for Faster Deconstruction of Cellulose
Source: PLoS One. 2016 Jan 28;11(1):e0147024. doi: 10.1371/journal.pone.0147024 (PMC4731210; doi:10.1371/journal.pone.0147024)
Supplement: S2 Table — Lower Ct values indicate more RNA transcript. (DOCX) [file pone.0147024.s006.docx]

S2 Table. Amount of RNA transcript in the ancestral *T. citrinoviride* population. Lower C_t_ values indicate more RNA transcript.

| Time (h) | C_t_ value*^a^* | | | | |
| --- | --- | --- | --- | --- | --- |
|  | *egl4* | *cbh1* | *bgl1* | *sar1* | *act* |
| 16 | 21.46 | 22.09 | 22.75 | 18.27 | 15.57 |
| 20 | 22.17 | 23.02 | 22.98 | 18.28 | 15.26 |
| 24 | **18.01** | 21.91 | 21.38 | 18.24 | 15.91 |
| 28 | 18.82 | 20.45 | 20.93 | 18.15 | 16.98 |
| 36 | 19.50 | 20.40 | 20.73 | 18.47 | 17.91 |
| 48 | **18.12** | **19.61** | **19.83** | 18.28 | 17.50 |
| 72 | 21.10 | 22.00 | 21.92 | 19.19 | 18.05 |

*^a^*C_t_ = threshold cycle, measures the amount of cellular RNA relative to control genes. C_t_ corresponds to the PCR cycle at which the fluorescent signal of the reporter dye crosses an arbitrarily placed threshold. Higher amounts of cellular RNA require fewer PCR cycles to reach this values, so lower C_t_ values indicate more cellular RNA. This C_t_ method assumes that the PCR efficiency of the target gene is similar to the internal control gene and that both are close to one. The genes *sar1* and *act* (both housekeeping genes in *Trichoderma*) serve as controls. The primers used in these experiments are in Table S7 below.
