# Supplementary material for: Experimental Evolution of Trichoderma citrinoviride for Faster Deconstruction of Cellulose
Source: PLoS One. 2016 Jan 28;11(1):e0147024. doi: 10.1371/journal.pone.0147024 (PMC4731210; doi:10.1371/journal.pone.0147024)
Supplement: S3 Table — Lower Ct values indicate more RNA transcript. (DOCX) [file pone.0147024.s007.docx]

**S3 Table. Amount of RNA transcript in the F1 selected population. Lower C_t_ values indicate more RNA transcript.**

| Time (h) | C_t_ value*^a^* | | | | |
| --- | --- | --- | --- | --- | --- |
|  | *egl4* | *cbh1* | *bgl1* | *sar1* | *act* |
| 16 | 22.63 | 21.58 | 21.95 | 19.04 | 16.11 |
| 20 | 17.96 | 22.07 | 21.54 | 18.91 | 16.01 |
| 24 | **13.82** | **13.08** | **17.02** | 17.96 | 16.87 |
| 28 | 15.01 | 13.59 | 17.85 | 18.11 | 16.45 |
| 36 | 18.71 | 17.81 | 20.86 | 19.26 | 17.77 |
| 48 | 18.12 | 19.61 | 19.83 | 18.28 | 17.50 |
| 72 | 21.10 | 22.00 | 21.92 | 19.19 | 18.05 |

*^a^*See footnote of S2 Table.
