# Supplementary material for: Experimental Evolution of Trichoderma citrinoviride for Faster Deconstruction of Cellulose
Source: PLoS One. 2016 Jan 28;11(1):e0147024. doi: 10.1371/journal.pone.0147024 (PMC4731210; doi:10.1371/journal.pone.0147024)
Supplement: S4 Table — Lower Ct values indicate more RNA transcript. (DOCX) [file pone.0147024.s008.docx]

**S4 Table. Amount of RNA transcript in the F4 selected population. Lower C_t_ values indicate more RNA transcript.**

| Time (h) | C_t_ value*^a^* | | | | |
| --- | --- | --- | --- | --- | --- |
|  | *egl4* | *cbh1* | *bgl1* | *sar1* | *act* |
| 16 | 20.45 | 21.59 | 22.16 | 18.36 | 15.38 |
| 20 | 20.05 | 21.64 | 21.92 | 18.39 | 15.94 |
| 24 | 17.86 | 20.01 | 20.27 | 17.94 | 15.82 |
| 28 | **14.12** | **10.91** | **17.48** | 17.78 | 17.49 |
| 36 | 17.95 | 14.88 | 20.15 | 18.96 | 17.14 |
| 48 | 21.31 | 21.61 | 22.36 | 19.27 | 18.45 |
| 72 | 21.19 | 21.30 | 21.71 | 19.42 | 19.20 |

*^a^*See footnote of S2 Table.
